# From moral treatment to modern music therapy: On the history of music therapy in Vienna (c. 1820–1960)

**DOI:** 10.1080/08098131.2018.1467481

**Published:** 2018-05-29

**Authors:** Andrea Korenjak

**Affiliations:** 1 Institute for the History of Art and Musicology, Department of Musicology, Austrian Academy of Sciences, Vienna, Austria; 2 Institute of Musicology, University of Vienna, Austria

**Keywords:** History of music therapy, medicine, psychiatry, Vienna

## Abstract

This article outlines the historical evolution and precursors of modern music therapy in Vienna from c. 1820 to 1960. The first section describes early attempts to purposefully integrate music into Viennese psychiatry and highlights the groundbreaking work of Bruno Goergen (1777–1842), who around the year 1820 deliberately incorporated music into psychiatric institutions based on his dedication to the ideals of “moral treatment” and “no-restraint” psychiatry. Shortly thereafter, the first medical dissertations on the therapeutic effect of music began to be published in Vienna. Around 1850, the emergence of an “active” form of “music therapy” (in the broadest sense) is recognizable; during the same time period, physicians began taking their patients’ musical preferences, education, and disposition into account. However, as university medicine became firmly rooted in the standards of experimental science, physicians increasingly lost their interest in music. The second section gives an overview of several cultural movements that emerged in the first half of the twentieth century and inspired the pioneers of music therapy in Vienna, paving the way for the foundation of modern Viennese music therapy in 1958. Among these were the life reform movement, the curative rhythm and dance movement, and anthroposophy, as well as psychotherapy and progressive music-educational concepts. At the conclusion of this article, the main traits of the history of music therapy in Vienna and some of the pervading premises of Viennese music therapy are summarized.

## Introduction

The entire secret is to be found in music, from which we want to construct a completely different therapeutic system.

Nicolaus Lenau (1802–1850), as quoted by his biographer Frankl (1854, p. 117)

The Vienna school of music therapy (*Wiener Schule der Musiktherapie*)^1^ 1The term “Wiener-Schule der Musiktherapie” appeared as early as 1960 (Sittner, 1960, p. 195); however, it was not commonly used at that time. It was later used by Viennese music therapists such as Editha Koffer-Ullrich (1904–1990) and Alfred Schmölz (1921–1995) to describe and establish their musical therapeutic approach. In this article, the term “Viennese music therapy” is alternatively used. is regarded as one of the pioneering music therapy schools in Europe (De Backer, Nöcker-Ribaupierre, & Sutton, 2014; Gold, 2003). With a history of 59 years of providing academic training as of 2018, Viennese music therapy defines its approach, in line with statutory legislation encapsulated in the Austrian Music Therapy Act, as an autonomous, research-based, artistic-creative, and expressive therapeutic method comprising different forms and techniques that share the “target-oriented application of musical means in a therapeutic relationship in order to restore, preserve, and advance psychic, physical, and mental health.”^2^ 2See the curriculum for the study of Music Therapy at the University for Music and the Performing Arts, Vienna, p. 2, URL: https://www.mdw.ac.at/upload/MDWeb/stdmp/downloads/Musiktherapie_16W.pdf (01.10.2016). The definition given here was taken from the Austrian Music Therapy Act and is not specific to the Viennese music therapy training course. This approach includes the deliberate therapeutic use of music’s functional effects (classified as activating or relaxing) as well as an awareness of the inherent relational potential of music and its ability to initiate social-communicative processes. Essentially, the Vienna school of music therapy is based on a tradition of teaching and practice that has been developed from clinical experience (particularly in pediatrics, psychiatry, and psychosomatics) and guided by humanistic and psychodynamic ideas (Stegemann & Fitzthum, 2016, p. 32).

The official history of the modern Vienna school of music therapy^3^ 3The term *modern* music therapy is used here to signify concepts starting around the middle of the twentieth century and to differentiate these from older approaches. begins with the foundation of the *Gesellschaft zur Förderung der Musikheilkunde* (Society for the Advancement of Music in the Healing Arts) on 26 November 1958 (see Mössler, 2008, p. 19).^4^ 4In the same year, the *Society for Music Therapy and Remedial Music*, later renamed *The British Society for Music Therapy*, was founded by the musician and music therapist Juliette Louise Alvin (1897–1982) and others (Mössler, 2008, p. 19). In 1959, the Vienna-born physician, neurologist, and psychotherapist, Hildebrand Richard Teirich (1907–1978), organized an international symposium on *Music in Medicine*, in Velden in the province of Carinthia (see also Teirich, 1958). In the same year, the first Austrian training course in music therapy was inaugurated at the Academy of Music and Performing Arts^5^ 5Today’s university of the same name. in Vienna (see Gold, 2003), headed by Editha Koffer-Ullrich (1904–1990). Although the official institutionalization of music therapy in Austria appears to be a relatively autonomous event, it should be understood in relation to historical precedents and to broader international developments in music therapy during this time period, particularly with regard to the USA and the United Kingdom.

The preconditions for the institutionalization of modern music therapy in Vienna in the second half of the 20th century, including the formative approaches of its pioneers, have been outlined chiefly by Fitzthum (2003a, 2003b, 2005, 2009, 2014), Mössler (2008, 2011, 2014), and Oberegelsbacher (1999, 2010), but, in spite of these valuable contributions, there is still a dearth of historical research in this area, particularly with regard to the first attempts to integrate music into the daily routines of 19th century Viennese psychiatric institutions (Korenjak, 2016). Apart from a few valuable publications, such as Kümmel (1977), Völkel (1979), Schumacher (1982), Kramer (van Xylander) (2000a, 2000b), and Schwartz (2012), the origins of the specified use of music in German and Austrian asylums in the late eighteenth and nineteenth century have been widely neglected.^6^ 6Among the most important publications devoted to the historical and cultural contexts of music and healing, the volumes edited by Schullian and Schoen (1948), Gouk (2000), and Horden (2000) will be highlighted here.


## Some preliminary remarks on the history of music therapy in Vienna

The history of music in medicine, psychiatry, and therapy in Vienna from c. 1820 to 1960 is discontinuous, but despite the gaps in its conceptual development, the idea that music can have a beneficial effect on the human soul and body was always present in common culture. Up until the middle of the twentieth century, there is no identifiable linear historical progression of music therapy in Vienna, but we can find the first clear attempts to integrate music into medicine and psychiatry as far back as the early nineteenth century.

Although the Viennese physician and poet Ernst von Feuchtersleben (1806–1849) emphasized that music was “rightfully praised as a means of psychic healing” (Feuchtersleben, 1845, p. 351), nineteenth-century Viennese medical dissertations used citations of older historical examples and legends, or the second-hand reports of other renowned physicians to explain music’s impact on patients; generally, claims made in these papers were not supported with systematically tried and tested clinical evidence. Thus, Joseph Denk (1796/97–1845), the author of one of the first Viennese medical dissertations on music, called himself an “inexperienced novice” who owed the credibility of his examples to other respectable sources because he could hardly draw on his own experience (Denk, 1822, p. 44). Subsequent Viennese dissertations on this subject, however, show the first traces of iatric consideration of the patient’s individual mindset based on clinical practice, taking into account both mental state and previous training in music (see below).

In each of the historical periods under examination in this article, we find the coexistence of different therapeutic concepts. Peter Lichtenthal’s (1780–1853) book *Der musikalische Arzt* (*The Musical Doctor*), published in Vienna in 1807, demonstrates how older paradigms overlapped with new musico-medical theories around the turn of the century. For instance, Lichtenthal equally draws on the baroque doctrine of the affects, as well as the concept of predefined forms of expression in specific musical modes (Lichtenthal, 1807, p. 95ff), and includes new insights evidently gained from empirical practice, such as considerations of the patient’s individual disposition and musical preferences (Lichtenthal, 1807, p. 172f).

Attempts to integrate music definitively into nineteenth-century Viennese psychiatric institutions were often based on dietetic principles (*res non naturales*): Music was believed to light up the soul (*Gemüth*) and to distract the mind from morbid thoughts. Particularly in private psychiatric institutions, such as Vienna’s Private Sanatorium for the Mentally Ill (*Privat-Heilanstalt für Gemüthskranke*), the privileged classes expected their cultivated lifestyle to continue for example through music lessons, literary readings, dance events, and other entertainments (see below). The Lower Austrian Provincial Institution for the Cure and Care of Mental and Nervous Diseases at Steinhof (*Niederösterreichischen Landes-Heil- und Pflegeanstalt für Geistes- und Nervenkranke “am Steinhof* *”*), established between 1904 and 1907 (today’s Otto-Wagner-Spital), also featured a music salon in the wing reserved for private patients. However, such early approaches were not defined as distinct music therapeutic methods. Not until the foundation of music therapy in 1957–1958, with a training course at the Vienna Academy of Music and Performing Arts, would it be possible for this particular therapeutic field to develop into a scholarly discipline that includes research and teaching.

By 1960, although music had already been integrated into some Viennese psychiatric institutions, the concept of Viennese music therapy was less influenced by previous psychiatric practice than by several cultural movements that took place in the first half of the twentieth century, such as the *Lebensreformbewegung*, the rhythm and dance movement, anthroposophy, reformed music education, as well as psychotherapy. In the section that follows, I will summarize the main historical preconditions and prominent figures during the period c. 1820 to 1960 that created and shaped the concept of music as therapy.

## Psychiatry and music in Vienna in the late eighteenth and ninteenth century

Up to the mid-eighteenth century, the mentally ill in Vienna were often kept in chains and barely clothed in the prison surroundings of the so-called *Militärstabstockhaus* (military staff house) at Neutor close to Salzgries. Occasionally, they were housed in the *Bürgerspital* (Citizen’s Hospital) founded in 1257, and although kept in a separate ward, they were still confined in cages (*Narrenkotter*). The Austrian physician Leopold Wittelshöfer (1818–1889) noted that the mentally ill were treated like beasts rather than human beings (see Wittelshöfer, 1856, p. 183). Medical care was not provided to these inmates because mental illness was generally regarded as incurable and caused by the individual’s own moral failings (see Wittelshöfer, 1856, p. 183; Viszánik, 1845, p. VII; Danz & Heinroth, 1812, p. 325).

The ideals of the Enlightenment, such as freedom, equality, and respect for the individual, led to a paradigm shift in the treatment of the mentally ill. Under the rule of the Austrian Emperor Joseph II (1741–1790), who dedicated himself to these ideals according to his own variation of enlightened absolutism, the *k. k. Irren-Anstalt* (Imperial and Royal Lunatic Asylum) – later infamously known as “Fools’ Tower” (*Narrenthurm*) – was erected as part of Vienna’s General Hospital. The establishment of such lunatic asylums by royal mandate in Austria should be seen in a broader international context, particularly with regard to the emergence of enlightened “modern psychiatry” and reform movements in France, Germany, and England. Vienna’s *k. k. Irren-Anstalt* was constructed as a circular building comprising 139 barred cells on 5 floors. However, the conditions in this asylum, considered to be Europe’s first institution exclusively for the mentally ill, were for the most part still inhumane (see e.g. Goergen, 1820; Mahir, 1846; Walber, 1844).

One of the most important advocates of the use of music in psychiatric institutions was Bruno Goergen (Görgen) (1777–1842), a native of Trier, who received his medical doctorate in Vienna in 1800 (see Korenjak, 2016, p. 188ff). After holding a position as assistant physician at the Vienna General Hospital, Goergen headed the *k. k. Irren-Anstalt* as its chief physician (from c. 1805 to 1808). In 1819, Goergen founded the first private sanatorium specifically for *Gemüthskranke* (patients suffering from mental illness) at house no. 173 in Gumpendorf, a well-to-do Viennese suburb at that time. In Goergen’s writings, the term *Gemüthskrankheit* (literally, “illness of the *Gemüth*”) was generally used with a varying range of meanings and signified a broad spectrum of mental illnesses, including “delusions” (the specific German notion of *Gemüth* is elusive and historically variable; in the Biedermeier period, the term *Gemüth* was increasingly identified with notions of the “soul,” “feelings,” and “sentiments”).^7^ 7The terms *Gemüth* and *Gemüthskrankheit* in their nineteenth-century context are detailed in Korenjak (forthcoming).


In his published clinical report, Goergen noted the existence of a large, beautiful, vaulted salon in his sanatorium designed for gatherings, carefully directed conversations, games, and especially for musical and literary presentations appropriate for *Gemüthskranke* (see Goergen, 1820, p. 13). In 1831, Goergen relocated his private sanatorium to Upper-Döbling, now in Vienna’s 19th district. Nicolaus Lenau, who according to Ludwig August Frankl had the vision of a new “music therapeutic system,” was one of the sanatorium’s most prominent patients. He died in Goergen’s clinic in 1850, then headed by Bruno Goergen’s son, Gustav.

In order to prevent patients from having “racing thoughts,” “feverish fantasies,” and “alienated sensibilities devoid of any harmony” (Goergen, 1820, p. 4), Goergen was convinced that the mind of the patient would need the “most salutary distraction,” while their body would benefit from occupation (Goergen, 1820, p. 15). Taking into account his patients’ desires, inclinations, age, and education, Goergen kept them mentally and physically engaged, alternating with music, educational lessons, conversation, games, gymnastics, and physical work in moderation.

The ideal state of the soul, the mind, and the emotions was generally seen as a “calmness of the mind and the soul [*Gemüth*]” accompanied by the absence of vivid feelings and desires (Lenhossék, 1825, p. 5). Mentally pleasant entertainments as well as appropriate amusement (*Unterhaltungslust*) were regarded as means to restore the balance of the mind and the *Gemüth*. In order to amuse his patients, Bruno Goergen would sometimes arrange small festive occasions with food, music, and dance. In this context, music and dance were not only believed to have a beneficial influence on the soul (*Gemüth*), they were also provided as a reward for patients’ “good behavior.” In cases of misconduct, however, Goergen excluded patients from these events, according to the principles of moral therapy. According to Georgen, physicians and caretakers had to act as “shining examples.” Georgen lived with his family in the sanatorium and used to share meals with his patients in order to teach them good manners.

The idea that the mentally ill could benefit from psychological care with a moral component characterizes the psychiatry of the Enlightenment and is reflected in terms such as *cura morale* (Chiarugi, 1793–94 ), *traitement moral* (Pinel, 1798), *psychische Curmethode* (Reil, 1803), or *moral management* (Haslam, 1817; Willis, 1823). Accordingly, the alienist was deemed a “moral guide” and “educator.” Despite Enlightenment progress, however, coercive measures continued to coexist with newer ideals, sometimes even justified by the principles of moral therapy.

## Music and medicine in Vienna

Bruno Goergen’s use of music in Vienna for the treatment of mental illness was pioneering for his time. In general, we can say that the relationship between music and medicine in eighteenth- and nineteenth-century Vienna was multifaceted and complex (see Korenjak, 2016, p. 182ff). Some Viennese physicians were themselves gifted musicians and music lovers. Among the most famous of these were the surgeon, violinist, and professor at the General Hospital Franz Schuh (1804–1865), and the renowned inventor of (medical) percussion Leopold von Auenbrugger (1722–1809), who was not only acquainted with the Mozart family but also with the librettist of Antonio Salieri’s 1781 comic opera *Der Rauchfangkehrer* (*The Chimney Sweep*). The founder of the Vienna school of pathological anatomy, Carl von Rokitansky (1804–1878), hosted a private music salon in the city. The Prussian-born Austrian surgeon and gifted amateur pianist, Theodor Billroth (1829–1894), was a close friend of Johannes Brahms (1833–1897) and of the influential music critic Eduard Hanslick (1825–1904).^8^ 8In his famous book *On the Musically Beautiful* (1st edition, 1854), Hanslick devotes a whole chapter to the “aesthetic comprehension of music,” which he differentiates sharply from “pathological listening.” Hanslick was also aware of music’s integration into psychiatry (Hanslick, 1854). Billroth was even consulted by Clara Schumann (1819–1896) concerning her son Ferdinand’s drug abuse. In 1895, Billroth’s book Wer ist musikalisch? (Who is Musically Talented?) was published posthumously by Hanslick (Billroth, 1895). However, musically talented physicians did not necessarily contemplate the use of music in medical treatment. Although many physicians mentioned music and its benefit for medicine, they did not develop the subject into even a rudimentary “therapeutic concept.”

There are, however, some Viennese medical dissertations written in the first half of the nineteenth century that do discuss the practical use of music in medicine and psychiatry (e.g. Denk, 1822; Bauer, 1837; Knöpfler, 1840; Popelka, 1845; Rosam, 1846; Hofgartner, 1847). The dissertations by Friedrich Carl Hofgartner and Hugo Kramoling give almost the same recommendations andinstructions for the application of music therapy (see Hofgartner, 1847, p. 47ff; Kramoling, 1847, p. 31f), which are as follows^9^: 9Their dissertations also predominantly reference the Hungarian physician Michael (Mihály) von Lenhossék (1773–1840).


(1) Music is particularly effective if it expresses the “natural language of the soul *(Gemüth*)” in a simple manner, especially in the case of those less educated.

(2) Music affects the unsound mind (*Gemüth*) favorably, particularly familiar folk songs and similar tunes.

(3) Music has to be in harmony with the patient’s individual state of sensitivity (*Empfindlichkeit*). Thus, for instance, it would be inappropriate for an erethistic (*erethisch*) person to be exposed to harsh or loud music.

(4) In order to stimulate the mind (*Gemüth*) beneficially, music must be introduced very slowly. The melancholic patient can be given solace by initial treatment with a dulcet adagio, followed by an andante. Towards the end of their session, a lively cheerful allegro should be played. To smooth an upset temper, however, one must begin with a loud allegro, gradually easing into a soft and calm melody (in the twentieth century, the question as to whether mood-congruent or mood-incongruent music should be used in treatment has been revisited).

(5) The musical instruments producing therapeutic music also have to be chosen by the physician in accordance with the patient’s preferences and habits. Thus, a melancholic person should not be exposed to the sound of the trombone or drum, since they may find the tonal aspects of these instruments “unbearable.” According to Hofgartner and Kramoling, only the flute and the harp would uplift a melancholic temperament (*Gemüth*). Furthermore, the physician has to be familiar with the specific qualities of different musical modes and the composition’s character.^10^ 10These aspects are treated extensively in Peter Lichtenthal’s book *The Musical Physician*.


(6) The physician should be aware that music affects well-educated people more strongly than the uneducated.

These recommendations are the initial indications of a paradigm shift from the general idea of music’s compelling (affective or mechanical) impact to an awareness of the patient’s individual perception of music based on their actual mood and disposition, musical preferences, previous musical training, etc. At the end of his dissertation, Hofgartner wonders at the neglect of music in contemporary medicine, “since medicine has enjoyed the most unbelievable progress in the last fifty years, not only in diagnostic but also in therapeutic respects” (Hofgartner, 1847, p. 49). For Hofgartner, the reason could be found in the “iatric ambition of the modern era,” which is “more than ever focused on finding remedies that are more generally applicable and involved with lower costs” (Hofgartner, 1847, p. 49). It can be said that this argument is still true today.

## Beginnings of music psychology

Although Wilhelm (Guilielmus) Knöpfler, in his medical dissertation *de Influxu Musicae in Corpus et Animum*, affirmed that music could be numbered among the most effective pharmacological mediums (Knöpfler, 1840, p. 31), as university medicine became oriented towards the standards of experimental science, physicians increasingly lost their interest in music, the effects of which defied scientific prediction and control at that time (Korenjak, 2016). This paradigm shift was particularly relevant in Vienna. Furthermore, the awareness that mental illness could be caused by a disease of the brain (e.g. Gall, 1829; Griesinger, 1845) was linked to the dominance of biologically oriented medicine and psychiatry. Nevertheless, considerations of music’s influence on the soul and body continued to be discussed in musico-philosophical and esthetic writings.

It is also noteworthy that the genesis of experimental music psychology in the second half of the nineteenth century was not guided by an interest in the “therapeutic” influence of music per se, but initially, more so by a search for music’s fundamental “elements” and “stimulus magnitudes” (these early approaches of psychophysics were founded for example by Fechner, 1860; Helmholtz, 1863). Later scientific discoveries in this field had an influence on modern music therapy and “music effect research.” Interestingly, scientific methodologies have assumed major importance in more recent research in music therapy (see e.g. Wheeler & Murphy, 2016).

An approach toward a more “integrative” and “holistic” perception of music was advanced by Richard Freiherr von Ehrenfels (1859–1932), born in the Vienna suburb of Rodaun (now the 23rd district). As a precursor of Gestalt psychology, Ehrenfels in his famous treatise *Ueber “Gestaltqualitäten”* (*On the “Principles of* gestalt*”*) (1890) criticized the scientific orientation toward pure “single elements” assumed in human (auditory) perception. Indeed, his concept of the “summative” (*Übersummenhaftigkeit*) points to the fact that the “whole” is something different than an aggregation of its single parts. Accordingly, a melody as a whole, its Gestalt quality, is *more* than the sum of its constituent single tones. For Ehrenfels, the perception of sound and music is initially based on the whole or the Gestalt (such as a melodic movement or a sound representing the sum of all its overtones); then in a *second* process, the elements of this perception (such as single tones of a triad or single overtones) can be identified. More importantly, the perception of music is not only based on musical structure but is also a result of the listener’s active “structuring” of the sounds (e.g. due to the listener’s musical training). Although Gestalt psychology did not influence modern Viennese music therapy directly, Ehrenfels’ thoughts were important for the awareness of an aesthetic dimension in music that goes *beyond* its purely physical effects.

## *Lebensreformbewegung* and “rhythmic movement”

One of the most famous photographs of Gustav Klimt (1862–1918) and Emilie Flöge (1874–1952) shows both of them in flowing garments symbolizing the contemporary idea of the “emancipation and liberation of bodily movement.” The so-called life reform movement (*Lebensreformbewegung*) emerged in the late nineteenth century, along with reconsiderations of diet (such as vegetarianism) and the concept of “natural medicine,” proposing a new (and anti-modernist) “way of living” as well as a “back-to-nature ideology” that featured such ideas as the liberation from bourgeois dress codes and dwelling spaces, physical exercise, fresh air, nudism, etc. At the beginning of the twentieth century, these reforms were often associated with aesthetic criteria that influenced clothing, body culture, gymnastics, and dance. Contemporaneously with the *Lebensreformbewegung* and the *Neue Tanzbewegung* (expressive dance movement), several modalities of (curative) rhythmic movement became popular: e.g. Jaques-Dalcroze’s (1865–1950) “eurhythmics,” Rudolf von Laban’s (1879–1958) “eukinetics,”^11^ 11In the sense of a “theory of the harmony of movement” (Laban, 1900, 1926, 1966/1976). anthroposophism pioneer Rudolf Steiner’s (1861–1925) “eurhythmy [*Eurythmie, sic*],” and Aleks Pontvik’s (1909–1979)^12^ 12Pontvik’s year of birth is uncertain. According to Wikipedia, he was born in 1909. “psycho-rhythmy [*Psychorhythmie*]” (see also Fitzthum, 2003a, 2005).

The Vienna-born Swiss music educationalist and composer Émile Jaques-Dalcroze (1865–1950) founded eurhythmics, an “elemental” music-motoric educational theory focusing on the physical experience of musical emotions expressed through movement (see Jaques-Dalcroze, 1917, 1921; Jaques-Dalcroze & Boepple, 1907a, 1907b). Fitzthum points out two of Jaques-Dalcroze’s pupils that were particularly important in the preliminary stages of modern Viennese music therapy: Rosalia Chladek (1905–1995), who founded the so-called Hellerau-Laxenberg School (1925–1938)^13^ 13In 1938, the *Anthroposophical Society* was prohibited by the National Socialists. and a dance company of the same name near Vienna that gave a new impetus to the rhythmical education of handicapped children; and Marie-Elisabeth (“Mimi”) Scheiblauer (1861–1968), who introduced music and eurhythmics to curative-educational programs for handicapped and deaf–blind persons by working for example with the patients’ sensation of vibration (see e.g. Scheiblauer, 1926, 1945, 1951, 1963). Besides psychiatry, the use of music and rhythmics for hospitalized children, as well as for developmentally challenged and disabled persons, was one of the first modern instances of the practical application of music for therapeutic purposes in Vienna (see e.g. Koffer-Ullrich, 1960, 1969; Weinhengst, 1965; Wesecky, 1963).

Hildebrand Teirich, one of the founders of music therapy, was himself inspired by Eugen Sutermeister (1862–1931), who had lost his hearing as a consequence of contracting meningitis at the age of four.^14^ 14I owe this reference to Beglinger (2010). Under the title *Lieder aus stiller Welt* (*Songs from a Silent World*), Sutermeister published an anthology of his poems in 1901. A short passage from his poem *Wie der taube Dichter hört* (*How the Deaf Poet Hears*) illustrates Sutermeister’s perception of sound and music:

[…] what feelings of pleasure slowly come over me,/I am carried and cradled by the tide in play, though never did I swim – /Surrendered to the waters and the winds in sweet rapture,/This is my inner awareness of hearing, this is my perception of sound. (my translation; Sutermeister, 1901, p. 14)

The concept of eurhythmy, introduced by Rudolf Steiner and Marie Steiner, neé Sievers, also had an inspirational influence on early modern music therapy in Vienna. The pioneers of music therapy in Vienna, such as Editha Koffer-Ullrich (1904–1990), Alfred Schmölz (1921–1995), and Ilse Castelliz (1914–2012), were familiar with Steiner’s anthroposophical system of thought. The musical instruments Steiner preferred, including pentatonic flutes and lyres, as well as the use of specific intervals, had a lasting effect on Viennese music therapy, which was founded several decades after the introduction of Steiner’s theories (see Oberegelsbacher, 2010; Fitzthum, 2003a, p. 47f).

According to Steiner, the human soul should not only find expression though the voice, but even more so through the *whole* human body, producing what he termed an “epiphany of the speaking soul” (Steiner, 1928) and a “visible utterance” or “visible song” (Steiner, 1927a, 1927b). Steiner’s concept was enhanced with music (tone eurhythmy), which relies on melody, harmony, and rhythm. Although not regarded as a “universal remedy,” remedial eurhythmy (*Heileurythmie*) was based on the idea of “healthy movements” that would beneficially affect the “unsound organism” (see Steiner, 1924, p. 53). Together with the gynecologist (Maria) Ita Wegman (1876–1943), Steiner founded the field of anthroposophic medicine. In 1924, the Russian dancer Olga Samyslowa (1895–1989) was commissioned by Marie Steiner to found a school for eurhythmics in Vienna.^15^ 15Later, in 1935, Trude Thetter (1907–1982) together with Margarethe Eckinger (1908–1993) established a *eurhythmic* training course. To the present day, e*urhythmics* continues to be taught in Vienna.


In the same year, the first Austrian Montessori school was also established in Vienna. According to Maria Montessori (1870–1952), one of the most important pedagogical endeavors is giving children an understanding of music. Montessori observed that small children would respond to music primarily through movement. Furthermore, she was convinced that music is not reserved for a chosen few. Everyone should be enabled to “enjoy this universal language of humankind and the expression of all the feelings of the soul as well” (Montessori, 2006, p. 20).

## Reform pedagogics, Carl and Gertrud Orff

In the famous Berlin Dada manifesto *Aufruf zur Elementaren Kunst* (*A Call for an Elemental Art*) (1921), art was claimed to be something deriving from its own internal elements and logic, rather than a philosophical pursuit. And in general, this “onset [Einbruch] of the elemental in art and education” (Preußner, 1963/64, p. 67) also inspired musicians and music teachers. Carl Orff (1895–1982), the most important and influential music pedagogue of the new generation, characterized “elemental music” as pre-intellectual, earthy (*erdnah*), natural, and physical; as always bound up with movement, dance, and language. According to Orff, music can be learned and enjoyed by anyone and has to be practiced *actively*, not only by listening, but also by participating in the performance (Orff, 1963/64, p. 16). Orff laid the groundwork for the musical education of children and laypeople through his pedagogical program known as the *Orff-Schulwerk*.^16^ 16The first edition of his *Elementare Musikübung* (*Elemental Musical Training*) was published between 1932 and 1935.


In Orff’s concept (similar to ideas found in eurhythmics and other related movements), music, language, movement, and dance are interrelated through rhythm (Orff, 1976, p. 17). The *Orff-Schulwerk* aims to support individual creativity and was conceived as a source for improvisation: “The Schulwerk, in each of its phases, was [always] intended to provide impulse to [further] self-initiated creations; thus, it will never be completed but will always be in progress, in a state of flux” (Orff, 1963/64, p. 13).

Carl Orff was pleasantly surprised when his *Schulwerk* was integrated into remedial educational curricula, for example by Karl Hofmarksrichter (1900–1976), head of the *Straubing School for the Deaf-Mute* in Germany (see also Orff, 1962; Wolfgart, 1971, 1975). In addition to Orff’s emphasis on active participation and improvisation (reflected in “active music therapy”), he gave further stimulus to music therapy through the creation of Orff instruments. To this day, Orff instruments and the *Orff-Schulwerk* program have been used within primary music education, as well as by many modern music therapists. Moreover, Gertrud Orff (1914–2000, née Willert), one of Carl Orff’s pupils and later his second wife, created the so-called Orff Music Therapy, combining the *Orff-Schulwerk* with a therapeutic approach geared particularly to the needs of handicapped and autistic children (see Orff, 1974, 1984, 1989). In 1973, the Institute for Social and Remedial Education was founded at the Mozarteum’s Orff-Institute in Salzburg.^17^ 17A course in *Music and Dance in Social Work and Integrative Education* is presently offered at the Mozarteum’s Orff-Institute (see Salmon, 2009).


Together with the Austrian–German gymnastics and dance educator Dorothee Günther (1896–1975), Carl Orff founded the Union for Applied and Free Movement (*Bund für angewandte und freie Bewegung e. V*.), also known as the *Günther-Schule* in Munich. Based on the beliefs of Emile Jaques-Dalcroze, Rudolf von Laban, and Elisabeth (“Bess”) Mensendieck (1864–1960), Günther’s concept comprised not only dance, musical-rhythmic physical education, and medical gymnastics but also singing, breathing, and voice exercises. One of Günther’s main ideals was the overcoming of repressed expression and creativity (Günther, 1929).

## Beyond “musical” and “unmusical”

Similar to Dorothee Günther, Heinrich Jacoby (1889–1964), in his book *Beyond* “Musical” *and* “Unmusical”*: The Liberation of Creative Forces Exemplified by Music*, assumed that there is no such thing as “a lack of talent,” but rather an “obstruction of musical self-fulfillment” (see e.g. Jacoby, 1921, 1924, 1925). Thus, Jacoby emphasized the importance of free musical improvisation and the creative process. The spontaneous playing of (piano) music (*spontanes Selbst-Erfinden*) and self-expression through music (Aus-dem-*Eigenen-Schöpfen*) were key ideas of his teaching principles (Jacoby, 1924, p. 39). Inspired by the Berlin gymnastics teacher Elsa Gindler (1885–1961) and based on individual psychology, Jacoby developed his musical didactic system in consultation with Leonhard Deutsch (1887–1952) and the Vienna-born Alfred Adler (1870–1937).

Oberegelsbacher (2010) identifies a direct connection between Jacoby’s (and Gindler’s) body of thought and Alfred Schmölz’s (1921–1995) approaches to music therapy, as demonstrated for example in his therapeutic concept of “practicing without practice” (*Üben ohne Übung*) (see Gathmann & Schmölz, 1994, p. 343).^18^ 18Alfred Schmölz headed academic training in music therapy in Vienna from 1970 to 1992. By encouraging creativity, according to Schmölz, music therapy is intended to (re)activate the potential for (self-)expression that may have become atrophied or obstructed in an individual, even if such recovery is only possible within the “developmental limits of the disability” (Schmölz, 1983, p. 255, 1974). The musical therapeutic approach is therefore not oriented toward a “final (artistic) result,” but rather toward a reflection on the process, the patient’s experience of his or her own creative potential in the context of music-related communication (Schmölz, 1974, p. 176; Gathmann & Schmölz, 1994). Schmölz’s framework, which has had an ongoing influence on modern Viennese music therapy, is closely related to the field of humanistic psychology emerging in the mid-1950s and its belief in the need for a creativity “*growing out of the uniqueness of the individual*” in order to “*actualize himself, to become his potentialities*” (Rogers, 1961, p. 350 and p. 351; italics in the original).

## Psychoanalysis and music

Despite his passion for Yvette Guilbert (1865–1944), Sigmund Freud (1856–1939) noted he was “almost unable to enjoy music” (Freud, 1914, p. 197). In contrast to the aspirations of the rhythm movement, the psychoanalytic method can be characterized as a pure “talking cure,” as Berta Pappenheim (Anna O.) fittingly phrased it (see Breuer & Freud, 1895, p. 23, *passim*). Nevertheless, psychoanalytic considerations of music and the unconscious appear in many writings by Freud’s followers (e.g. Graf, 1910; Teller, 1917; Mosonyi, 1935; [Sigmund] Pfeifer, 1923; Sterba, 1939, 1946; Kohut & Levarie, 1950; Ehrenzweig, 1953, 1960; Haisch, 1953, 1954; Kris, 1952, 1977; Kohut, 1951, 1956, 1957; and others).

The theoretical development of modern Viennese music therapy has often drawn from depth psychology, incorporating ideas such as the existence of an unconscious that could be expressed and “revealed” through musical activity. Moreover, the music therapeutic relationship between the client/patient and the therapist has frequently been designated in psychoanalytical terms. Karin Mössler points out that the psychotherapeutic approach (including psychoanalytic, psychodynamic, and humanistic concepts) has been the “leading paradigm” (Mössler, 2011, p. 158ff.) in music therapy.

The rise of National Socialism forced not only Freud, but also Sterba, Ehrenzweig, Kris, and Kohut to flee from Vienna. Pfeifer died 1945 in the Buchenwald concentration camp. Other Jewish thinkers and seminal figures in music and therapy, who succeeded in fleeing the Nazi terror, continued their work abroad, predominantly in the USA. One Jewish refugee who became a pioneer in music therapy was the Vienna-born pianist and composer Vally Weigl (1894–1982), whose merits were extolled by Elena Fitzthum in her edited book *Give them Music. Music Therapy in Exile: The Story of Vally Weigl* (Fitzthum, 2003b; see also, 2003a).

## Harmonics

From its beginnings, Viennese music therapy was also indirectly inspired by Carl Gustav Jung’s (1875–1961) notion of archetypes and, more importantly, by Hans Kayser’s (1891–1964) “doctrine of harmonics” (*Harmonik*) (Jung, 1997; Kayser, 1947; Timmermann, 2009, p. 181). The Swedish music therapist Aleks Pontvik (1909–1979), himself a follower of Jung and Kayser, created the concept of musical archetypes, which he claimed to have discovered in Johann Sebastian Bach’s music (Pontvik, 1948, 1955). Similar to Steiner, Kayser, and others who were inspired by Pythagorean-Platonic thinking,^19^ 19For the Pythagoreans and Plato, music’s ability to (re)create “harmony” is crucial for well-being and health. According to Plato, the muses bestowed upon humans a harmony containing “movements,” which are related to the “movements of the soul” (Plato, 1992, p. 73). Thus, music can restore order in the movements of the soul when they have become unbalanced. Heaven and the soul, which according to Plato were created simultaneously, find their profound union in cosmic harmony, which is expressed in numbers (see Korenjak, 2003). Pontvik was convinced that “well-structured” and harmonious “impulses” (e.g. sounds played on a monochord or “psycho-chord,” respectively) would have a salutary effect on the soul. Pontvik called his approach *Psychorhythmie* (Pontvik, 1954, 1962). Hans Sittner (1903–1990), president of the Vienna Academy of Music and Performing Arts, extended an invitation to Kayser to give a series of lectures in Vienna, but Kayser did not accept his offer (Fitzthum, 2014, p. 28). Eventually Rudolf Haase (1920–2013), Kayser’s student, did come to the Vienna academy to continue the (speculative) search for harmonic universals. The doctrine of harmonics was originally integrated into music therapy training as a “conceptual foundation” of Viennese music therapy (Joham, 2000, p. 17), but it has lost significance in subsequent years.

## The beginning of modern music therapy training in Vienna

In 1952, Hans Sittner became acquainted with music therapy while in the USA, and after his return, he contacted Hans Hoff (1897–1969), head of the Psychiatric-Neurologic University Clinic in Vienna (Schmölz, 1972). Later, the Viennese violinist Editha Koffer-Ullrich (1904–1990) also explored music therapeutic approaches in the USA (Feichter, 2016, p. 43). Koffer-Ullrich visited East Coast hospitals where music therapy was provided for soldiers traumatized by war (Stegemann, 2016). After her return from the United States, Koffer-Ullrich headed the first training course in Vienna from 1959 to 1970. Additionally, in 1966 the pediatrician Andreas Rett (1924–1997), whom Rett syndrome is named after, advocated music therapeutic work with handicapped children in Vienna (see e.g. Rett, Grasemann, & Wesecky, 1981).

In the early stages, Viennese music therapy was based on an interdisciplinary collaboration of physicians, psychologists, musicians, pedagogues, and scientists (see Schmölz, 1972, p. 408). The first “music therapists” in Vienna were autodidacts (see Rett & Wesecky, 1975, p. 193; Rett, 1973, p. 187) regarded as auxiliary personnel and “physicians’ assistants” (Koffer-Ullrich, 1960). Although inspired by the empirical, clinically oriented music therapy in the USA, and influenced by early music therapy practitioners (such as Teirich and Pontvik), in Vienna, according to Schmölz, an attempt was made to blaze an “autonomous path for therapy and training” (Schmölz, 1972, p. 409). However, music therapy’s historical precedents in Vienna were reflected until recently in the organizational structure of the Institute for Musical and Physical Education and Music Therapy at the University of Vienna, which comprised three departments: Musical and Physical Education, Music Therapy, and Integrative Education in Breathing, Voice, and Movement. In 2016, the Department of Music Therapy was transformed into an independent institute.

## Conclusion

Bruno Goergen’s pioneering integration of music into Viennese psychiatry was continued by his successors, among them his son, the physician Gustav Goergen (1814–1860), and in particular by the Austrian neurologist and psychiatrist Heinrich Obersteiner Junior (1847–1922). It is important to realize, however, that early attempts to integrate music into psychiatry did not rely on a “therapeutic process,” or on the idea of the therapeutic expression of the self through music. Music was primarily seen as a means of distraction (e.g. from fixed ideas), or of education (in the form of music lessons) and entertainment (by means of asylum concerts and chamber music). Accordingly, the Viennese psychiatrist Alexander Pilcz (1871–1954) concluded in 1905:

[…] none of the elements of music, [such as] melody, harmony, rhythm, instrumental timbre, inherently possess any specific properties relating to mental illnesses, and indeed, music offers no other benefit than distraction and entertainment, which modern care of the insane makes use of in as many ways as possible. (Pilcz, 1905, pp. 8–9)

Even though by 1960 Vienna was able to look back upon a long tradition of the use of music in psychiatry, the very early fundament of the modern Vienna school of music therapy was built on anthroposophy, harmonics, and progressive education (Mössler, 2008). However, the idea that those who had become mentally imbalanced could only be restored by means of harmonious sounds and certain intervals was replaced by the notion of every individual’s creative potential (Mössler, 2014).

The impulses derived from twentieth-century reform movements, including progressive music education (self-expression and improvisation), had a stronger influence on modern therapeutic concepts than nineteenth-century Viennese psychiatry. Unlike nineteenth-century practice, the therapeutic relationship was discovered to be a crucial part of the therapeutic triad (patient–music–therapist) and the “musical dialogue.” From today’s perspective, clinical insight, the humanistic approach, and inspiration from psychotherapy are considered to be the main influences on Viennese music therapy training (Stegemann & Fitzthum, 2016, p. 32).

In summary, the foundations of the Vienna school of music therapy are multifaceted and to a great degree interdisciplinary. Emerging from an environment shaped by several mid-twentieth-century reform movements (in dance, rhythmics, music pedagogy, remedial education, psychotherapy, and psychiatry), modern Viennese music therapy operates according to the basic understanding that music is a creative expression of the self, which can be enjoyed by anyone, irrespective of and *beyond* any claims to artistic professionalism. Music therapy is based on the creative process and on improvisation – processes whose importance extends *beyond* expected results – and is able to facilitate a musical dialog and communication that goes *beyond* words. Thus, music is supposed to (re-)establish social interactions and relationships, and to further insights into the self *beyond* any mental or physical illness. Based on research and teaching, Viennese music therapy encompasses the prevention, treatment, and rehabilitation of acute and chronic illnesses, as well as the advancement of social competence.^20^ 20See http://www.mdw.ac.at/I113/html/mth/?PageId=3192.

